# Annular elastolytic giant cell granuloma: an unusual presentation in non-sun-exposed areas^☆^

**DOI:** 10.1016/j.abd.2022.02.010

**Published:** 2023-06-29

**Authors:** Claudia Suárez, Gonzalo Hevia, Catalina Silva-Hirschberg, Alex Castro

**Affiliations:** a Department of Dermatology, Hospital Padre Hurtado, Santiago, Chile; b Department of Dermatology, Facultad de Medicina, Clínica Alemana, Universidad del Desarrollo, Santiago, Chile; c Department of Pathology, Facultad de Medicina, Clínica Alemana, Universidad del Desarrollo, Santiago, Chile

Dear Editor,

A 76-year-old man with a prior diagnosis of type 2 diabetes mellitus and hypertension consulted our Dermatology practice with a 20-year history of progressive pruritic lesions. He had been previously treated with topical antifungals and corticosteroids without response.

Physical examination revealed multiple, large, well-circumscribed annular plaques on the trunk, back and arms. Lesions showed central healing surrounded by a red inflammatory zone and a palpable erythematous border on the extreme periphery of the plaques. Post-inflammatory hyperpigmentation was also noted (Fig. 1A and 1 B). Dermoscopy on the periphery of the lesions showed unstructured orange zones on an erythematous background, with some whitish areas and fine vessels on the surface (Fig. 1C).

**Figure 1:**
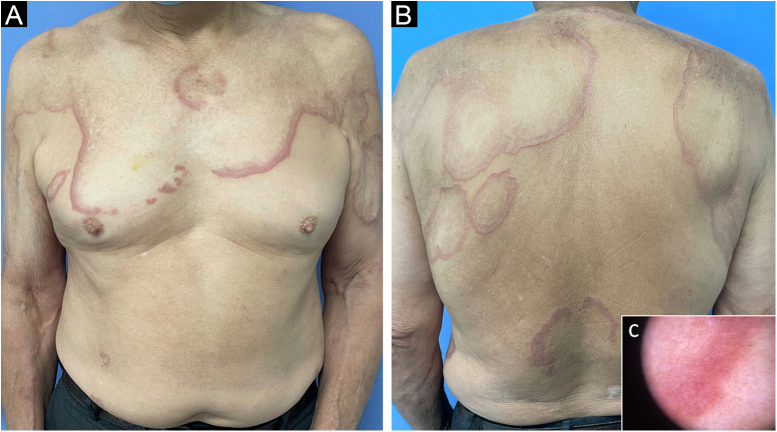
Annular plaques on the trunk and arms; anterior (A) and posterior view (B). Dermoscopy (magnification 20×) showing unstructured orange zones on an erythematous background (C)

A cutaneous biopsy of the lesion border was performed; histopathological analysis on the H-E stain showed multiple foci of histiocytic and multinucleated giant cells and areas of collagen degeneration on the dermis (Fig. 2A and 2 B). No increase in interstitial mucin was observed (Fig. 3A). Verhoeff's Van Gieson stain showed elastoclasia and loss of elastic fibers in the foci of histiocytic infiltrate (Fig. 3B and 3 C). Given the clinical presentation and histopathological analysis, Annular Elastolytic Giant Cell Granuloma (AEGCG) was diagnosed.

**Figure 2:**
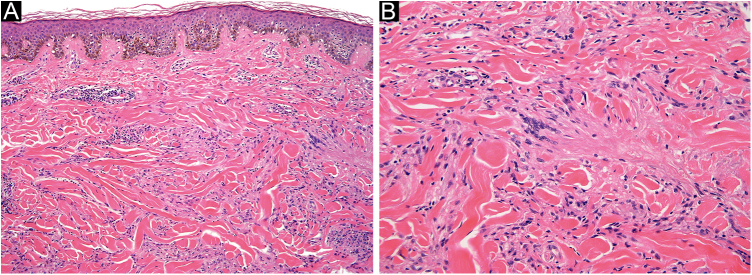
(A) Hematoxylin & eosin, 100×; perivascular and interstitial infiltrate with lymphocytes and numerous histiocytes. Collagen degeneration areas surrounded by multiple histiocytes and multinucleated giant cells. (B) Hematoxylin & eosin, 200×; collagen degeneration on the dermis

**Figure 3:**
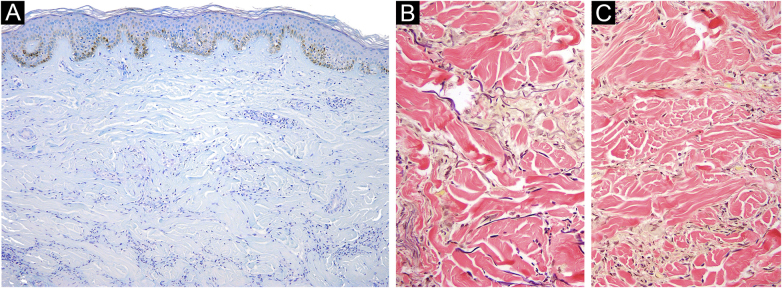
(A) Alcian blue, 100×; absence of mucin. (B) Verhoeff-Van Gieson elastic, 200×; elastophagocytosis and elastoclasia in the active border of a plaque. (C) Verhoeff-Van Gieson elastic, 200×; absence of elastic fibers in the central area of the same plaque

AEGCG is a rare cutaneous granulomatous disease of unknown etiology. It was first described by O'Brien in 1975 as a variant of granuloma annulare located in photo-exposed areas.^1^ However, it is now considered a distinct entity given its characteristic histopathological findings: non-palisading granulomas in the superficial dermis, abundant multinucleated giant cells, marked elastophagocytosis, and absence of mucin and necrobiosis.^2^

It has been proposed that factors such as ultraviolet radiation, heat, and vascular damage could generate elastolysis and an antigenic change of the elastic fibers, triggering a cellular immune response and a consequent granulomatous reaction.^1^, ^3^ AEGCG has been associated with diabetes mellitus, hyperlipidemia, hypertension, vascular occlusion, arthritis and hematological and solid organ malignancies.^4^

Clinically, it is characterized by photo-distributed, slow-growing annular papules or plaques with erythematous borders and a slightly hypopigmented or atrophic center.^1^ Papular, reticular forms, and variants that involve non-exposed areas -as in our case- have been described.^3^ Dermoscopically, unstructured yellow-orange areas and desquamation in the periphery have been reported, with homogeneous reticular vessels in the center of the lesion.^5^

We examined our patient and comorbidities were not found, age-appropriate cancer screening was negative. Because our patient was an underground mining worker for over 30 years, we hypothesize that the unusual distribution of his lesions may be explained by heat rather than sunlight exposure.

The differential diagnosis includes other granulomatous and annular diseases such as tinea corporis, leprosy, tuberculosis, sarcoidosis, granuloma annulare and necrobiosis lipoidica.^6^ The histopathological analysis is crucial for a correct diagnosis. Mycotic and mycobacterial cultures may also be necessary to exclude infectious diseases.

We present a case of AEGCG in non-sun-exposed areas, which had been misdiagnosed and mistreated for decades. AEGCG is a rare disease that must be included in the differential diagnosis of annular plaques, in which a biopsy is critical to exclude other autoimmune and infectious diseases.

## Financial support

None declared.

## Authors' contributions

Claudia Suárez: Approval of the final version of the manuscript; intellectual participation in propaedeutic and/or therapeutic management of studied cases; manuscript critical review; preparation and writing of the manuscript.

Gonzalo Hevia: Approval of the final version of the manuscript; critical literature review; manuscript critical review; preparation and writing of the manuscript.

Catalina Silva-Hirschberg: Approval of the final version of the manuscript; critical literature review; manuscript critical review; preparation and writing of the manuscript.

Alex Castro: Approval of the final version of the manuscript; manuscript critical review; preparation and writing of the manuscript.

## Conflicts of interest

None declared.
